# ModFus-PD: synergizing cross-modal attention and contrastive learning for enhanced multimodal diagnosis of Parkinson’s disease

**DOI:** 10.3389/fncom.2025.1604399

**Published:** 2025-07-16

**Authors:** Xiangze Teng, Xiang Li, Benzheng Wei

**Affiliations:** ^1^Center for Medical Artificial Intelligence, Shandong University of Traditional Chinese Medicine, Qingdao, China; ^2^Qingdao Academy of Chinese Medical Sciences, Shandong University of Traditional Chinese Medicine, Qingdao, China; ^3^Qingdao Key Laboratory of Artificial Intelligence Technology for Chinese Medicine, Qingdao, China

**Keywords:** early diagnosis of Parkinson’s disease, multimodal representation learning, crossmodal attention, contrastive learning, multimodal fusion

## Abstract

Parkinson’s disease (PD) is a complex neurodegenerative disorder characterized by a high rate of misdiagnosis, underscoring the critical importance of early and accurate diagnosis. Although existing computer-aided diagnostic systems integrate clinical assessment scales with neuroimaging data, they typically rely on superficial feature concatenation, which fails to capture the deep inter-modal dependencies essential for effective multimodal fusion. To address this limitation, we propose ModFus-PD, Contrastive learning effectively aligns heterogeneous modalities such as imaging and clinical text, while the cross-modal attention mechanism further exploits semantic interactions between them to enhance feature fusion. The framework comprises three key components: (1) a contrastive learning-based feature alignment module that projects MRI data and clinical text prompts into a unified embedding space via pretrained image and text encoders; (2) a bidirectional cross-modal attention module in which textual semantics guide MRI feature refinement for improved sensitivity to PD-related brain regions, while MRI features simultaneously enhance the contextual understanding of clinical text; (3) a hierarchical classification module that integrates the fused representations through two fully connected layers to produce final PD classification probabilities. Experiments on the PPMI dataset demonstrate the superior performance of ModFus-PD, achieving an accuracy of 0.903, AUC of 0.892, and F1 score of 0.840, surpassing several state-of-the-art baselines. These results validate the effectiveness of our cross-modal fusion strategy, which enables interpretable and reliable diagnostic support, holding promise for future clinical translation.

## 1 Introduction

Parkinson’s disease (PD) is a complex neurodegenerative disorder that can lead to disability and substantially elevate the risk of developing dementia (Kalia and Lang, 2015), thereby severely impairing patients’ quality of life. By 2040, the global prevalence of PD is projected to surpass 12 million individuals (Zhu et al., 2024), making it the most rapidly increasing neurodegenerative condition worldwide (Gbd 2016 Parkinson’s Disease Collaborators, 2018). As no definitive cure exists, early intervention is essential to delay progression and improve outcomes (Becker et al., 2002). Studies report an average diagnostic delay of about 1 year after symptom onset. Economically, PD imposes a substantial burden, with annual costs in the U.S. exceeding USD 52 billion, including both direct medical and indirect societal expenses (Breen et al., 2013).

Motor symptoms are primary clinical indicators for PD diagnosis (Sveinbjornsdottir, 2016), but they often emerge when the disease has already advanced (Sveinbjornsdottir, 2016). However, by the time such symptoms become apparent, patients are often already in an advanced and irreversible stage. Early motor signs are typically subtle and nonspecific, often misattributed to aging or other conditions. For example, bradykinesia may present as vague complaints like “slowed movements” or “poor coordination,” which may go unrecognized (Bidesi et al., 2021; Höglinger et al., 2024; Jankovic, 2008). Non-motor symptoms have also gained attention as early diagnostic markers. Some, such as olfactory loss, may precede motor signs by years. Monitoring prodromal non-motor symptoms—like sleep disturbances, cognitive decline, anxiety, and depression—can enhance early detection of PD (Herz and Brown, 2023; Maggi et al., 2021; Stefani and Högl, 2020).

Despite their clinical utility, both motor and non-motor symptoms are typically assessed using rating scales, clinical scale assessments for Parkinson’s disease are often influenced by patients’ personal perceptions, emotional states, or recall bias, while clinician ratings may vary due to differences in experience, evaluation criteria, and assessment environments.

These limitations compromise the objectivity and reliability of assessments. To address this issue, researchers have increasingly turned to more objective neuroimaging techniques. In PD, distinct structural and functional brain abnormalities linked to disease pathology can be detected through imaging. Several studies have utilized neuroimaging data to characterize the brain alterations associated with PD, aiming to extract more objective biomarkers of neurodegeneration. Nevertheless, such structural brain changes in the early stages of PD are often subtle, and their imaging profiles may resemble those of other neurodegenerative disorders (Boonstra et al., 2021), complicating differential diagnosis.

Clinical rating scales offer rich representations of both motor and non-motor symptoms, while MRI provides objective imaging evidence of underlying neuropathology. To leverage the complementary strengths of these two modalities, prior studies have explored their integration. A central challenge lies in how to effectively fuse imaging and non-imaging data for accurate PD diagnosis. Existing multimodal fusion approaches often rely on simple feature concatenation, without adequately capturing their synergistic potential. In particular, they tend to overlook a critical aspect of multimodal learning—cross-modal interactions and the deep complementary nature of heterogeneous information sources (Chen et al., 2019). This study addresses the challenge of heterogeneous data alignment through multimodal representation learning and employs a cross-modal interaction module to analyze inter-modal relationships.

In fact, numerous studies have demonstrated strong associations between clinical assessments and neuroimaging findings, suggesting that the two modalities can jointly reflect disease characteristics. For instance, Hanafi et al. (2024) observed positive correlations between UPDRS scores and regional brain activity during gait tasks in PD patients. Yoo et al. (2024) conducted a longitudinal study spanning over 5 years, documenting UPDRS scores and MRI data, and reported that changes in motor-related brain regions were significantly associated with UPDRS ratings. Imaging and non-imaging data capture PD-related pathological features from different perspectives, offering complementary insights that can enhance diagnostic accuracy and robustness. Nevertheless, how to effectively model cross-modal interactions to fully exploit this complementarity remains an open research problem.

To this end, we propose a diagnostic framework for Parkinson’s disease, termed ModFus-PD, which integrates multimodal representation learning with cross-modal attention to leverage both clinical scale data and neuroimaging data for improved diagnostic performance. The framework is composed of three modules: multimodal representation learning, modality interaction, and classification. Specifically, the multimodal representation learning module employs a large language model to transform structured clinical rating scale scores into semantically enriched textual prompts, enhancing the interpretability of numerical assessments and making them compatible with a text encoder. In parallel with MRI inputs, a contrastive learning strategy is adopted to pretrain the image and text encoders, aligning the representations of both modalities within a shared latent space. Subsequently, the modality interaction module applies a cross-modal attention mechanism to integrate information from text and imaging features. The resulting fused features are then used for final classification. The main contributions of this study are as follows:

(1) This study introduces a novel diagnostic framework, ModFus-PD, in which the core multimodal representation learning module leverages contrastive learning to align deep feature representations from MRI data and clinical rating scale–derived textual embeddings. This alignment reduces cross-modal discrepancies and enables the effective integration of complementary features, facilitating accurate and synergistic information fusion for Parkinson’s disease diagnosis.

(2) We designed a structured clinical scale–to–text conversion module, which transforms numerical information from clinical rating scales into semantically enriched textual descriptions. This process enhances the semantic representation of clinical assessments and enables their effective integration into multimodal semantic alignment analysis.

(3) The model was evaluated on the PPMI dataset, achieving an accuracy of 90.3%, an AUC of 0.89, and an F1 score of 0.84.

The remainder of this paper is organized as follows: section 2 reviews related work on the diagnosis and progression of Parkinson’s disease. section 3 introduces the dataset and preprocessing procedures, and provides a detailed description of the core components of the proposed algorithm. Section 4 presents four experiments and evaluates both the overall model performance and the impact of modality selection on downstream tasks. Section 5 discusses the significance and limitations of this study. Section 6 concludes the paper by summarizing the main findings.

## 2 Related works

Clinical rating scales are essential tools for assessing the severity and progression of Parkinson’s disease (PD). Among these, the Movement Disorder Society–Unified Parkinson’s Disease Rating Scale (MDS-UPDRS) is currently the most widely adopted clinical instrument (Goetz et al., 2008). In addition, the State-Trait Anxiety Inventory (STAI), Epworth Sleepiness Scale (ESS), and Clock Drawing Test (CDT) are also frequently employed in PD evaluation (Hazan et al., 2018; Knowles and Olatunji, 2020; Walker et al., 2020). Several studies have explored the use of clinical rating scale data in combination with machine learning techniques to distinguish PD patients from healthy controls. For example, Martinez-Eguiluz et al. (2023) collected non-motor symptom data (e.g., autonomic function scores, and olfactory assessments) and evaluated nine classification algorithms, including support vector machines (SVMs) and multilayer perceptrons (MLPs). All models achieved classification accuracies exceeding 80%, with the best performance (SVM) reaching approximately 86.3%. However, scale-based assessments are not without limitations. Their outcomes can be influenced by patients’ subjective perceptions, demographic factors such as age and educational background, the evaluator’s clinical experience, environmental conditions, and inherent subjectivity in scoring criteria.

Compared to clinical rating scales, medical imaging offers more objective and quantifiable biomarkers, serving as a complementary diagnostic modality for Parkinson’s disease (PD). These imaging techniques include structural MRI, Diffusion Tensor Imaging (DTI), and functional radionuclide imaging methods such as PET and SPECT. Zheng et al. (2024) employed a deep neural network trained via contrastive learning to analyze brain MRIs from hundreds of PD patients and healthy controls, revealing PD-specific neuroanatomical patterns that effectively distinguish patients from normal individuals. Khachnaoui et al. (2022) applied three machine learning algorithms to differentiate PD patients from healthy controls within the Scan Without Evidence of Dopaminergic Deficit (SWEDD) cohort, with hierarchical clustering achieving the highest accuracy, sensitivity, and specificity.

In recent years, multimodal deep learning approaches have significantly advanced the field of medical artificial intelligence by learning joint feature representations from heterogeneous data sources, thereby enhancing diagnostic performance and model interpretability. Integrating clinical assessments with neuroimaging data allows for the combination of symptom-level information and objective biological markers to improve diagnostic accuracy. For example, Yang et al. (2021) proposed a two-layer ensemble learning model that integrates imaging features from MRI and DTI with clinical evaluation scores to classify Parkinson’s disease (PD) patients. The model achieved an accuracy of 96.9% under 10-fold cross-validation, substantially outperforming models based on either clinical or imaging data alone. Beyond diagnostic classification, clinical score–imaging fusion has also been applied in prognostic modeling.

Multimodal deep learning, which integrates feature representations from heterogeneous data sources, is emerging as a prominent research focus in medical artificial intelligence. In the diagnosis of Parkinson’s disease and other neurological disorders, researchers have developed various multimodal representation learning frameworks to leverage complementary information across modalities, thereby enhancing diagnostic accuracy and model interpretability. Heidarivincheh et al. (2021) proposed MCPD-Net, a variational autoencoder (VAE)-based architecture that fuses visual silhouette and accelerometer data for PD classification. The model maintains robustness under missing modality conditions and achieved an F1 score improvement of 0.25 over vision-only models and 0.09 over other multimodal approaches. Chen et al. (2023) introduced the OLFG model, which integrates MRI, PET, and clinical data via orthogonal latent space projection and incorporates a feature weighting matrix along with cross-modal graph regularization to capture inter-modality relationships. The method outperformed existing approaches on the ADNI dataset, demonstrating its diagnostic utility in Alzheimer’s disease (AD) classification.

Although existing multimodal approaches have achieved notable improvements in diagnostic performance, their modality fusion strategies still leave room for enhancement. Many current methods rely on shallow fusion techniques—such as simple feature concatenation—where features from different modalities are directly combined for classification tasks. Such strategies often overlook the complex, underlying interactions across modalities. The insufficient modeling of inter-modality complementarity and correlation may constrain the representational capacity of the fused features, thereby limiting performance gains in more challenging diagnostic scenarios. Therefore, developing more effective cross-modal interaction mechanisms to capture synergistic relationships between modalities remains a critical direction in multimodal research for Parkinson’s disease diagnosis.

## 3 Materials and methods

### 3.1 Data collection and preprocessing

This study utilizes the Parkinson’s Progression Markers Initiative (PPMI) database as the primary data source. Initiated by the Michael J. Fox Foundation in 2010, the PPMI aims to bring together researchers, medical professionals, and patients to advance the understanding of Parkinson’s disease (PD). From the PPMI database, we collected both imaging data and four distinct clinical assessment scales covering motor function (MDS-UPDRS), anxiety (STAI), sleepiness (ESS), and cognitive ability (CDT). Detailed information is provided in Table 1. All MRI scans were acquired using a 3T Siemens scanner with an MPRAGE sequence. A total of 383 participants were included, comprising 234 individuals with PD and 149 healthy controls. Inclusion criteria for PD subjects were as follows: (1) no prior pharmacological treatment; (2) availability of MRI scans and complete data for all four clinical assessments; and (3) age and sex matched with the healthy control group. All participants were between 45 and 80 years of age.

**TABLE 1:** Description of the clinical assessment scales used in this study.

Clinical assessment scales	Description
MDS-UPDRS	The movement disorder society-unified Parkinson’s disease rating Scale is an internationally recognized scale for assessing PD, used to comprehensively evaluate both motor and non-motor symptoms of PD. This study primarily utilizes the motor symptoms section.
ESS	The Epworth sleepiness scale is a standardized questionnaire used to assess daytime sleepiness, reflecting the patient’s sleep quality.
STAI	The state-Trait Anxiety Inventory measures anxiety levels, assessing both emotional state and long-term anxiety traits.
CDT	The clock drawing test evaluates cognitive function, primarily reflecting executive function, visuospatial ability, and memory.

In this study, T1-weighted MRI scans underwent a series of preprocessing steps. First, AC-PC alignment was performed using the SPM12 toolbox, with manual adjustment of the image origin to the anterior commissure. Next, non-brain tissues such as the neck were removed using the built-in recon-all command in FreeSurfer, retaining only brain regions. Finally, all MRI scans were nonlinearly registered to the MNI152 standard space using the Advanced Normalization Tools (ANTs) software. We did not apply further intensity normalization or denoising to maintain the original MRI contrast patterns for downstream analysis.

### 3.2 Overview of our framework ModFus-PD

**FIGURE 1:**
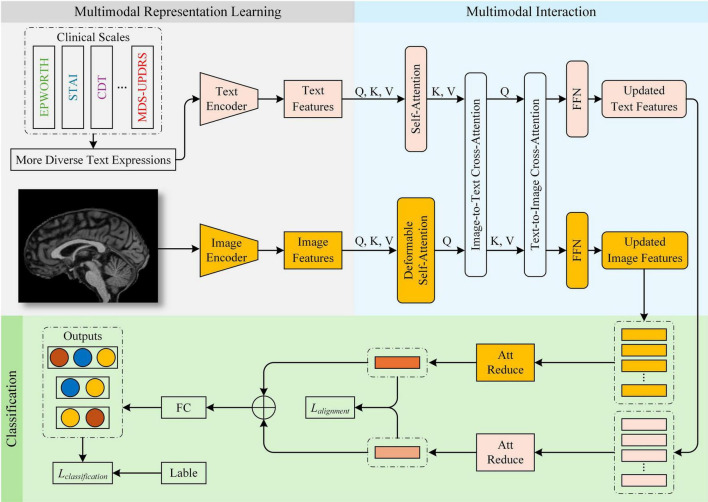
Overview of the ModFus-PD framework.

In this study, we propose a Parkinson’s disease (PD) diagnostic framework, ModFus-PD, which integrates multimodal representation learning with a cross-modal attention mechanism. The framework consists of three key components: (1) a multimodal representation learning (section 3.3), which includes medical prompt generation and contrastive learning to extract features from MRI scans and textual descriptions of clinical rating scores, and to align their representations in a shared latent space; (2) a multimodal interaction (MI) module (section 3.4), which enhances the semantic interaction between imaging and textual modalities through a cross-modal attention mechanism; (3) a hierarchical classification module (section 3.5), which aggregates the fused features for final disease prediction. In the Multimodal Representation Learning module, scale-derived text and MRI data are aligned using pre-trained text and image encoders. In the multimodal interaction module, semantic correlations between modalities are further strengthened. Upon obtaining the processed image and text features, the fused multimodal representation is fed into the classification module for PD diagnosis. An overview of the framework is presented in Figure 1.

### 3.3 Multimodal representation learning

Integrating MRI images with clinical rating scale data poses several challenges due to their inherent differences in data format, statistical distribution, and information representation. MRI data are high-dimensional and continuous, capturing spatial anatomical structures, whereas clinical scale scores are low-dimensional, discrete, and structured, representing subjective symptom assessments. These disparities lead to a substantial distributional gap and semantic mismatch between the two modalities. Specifically, MRI reflects objective anatomical alterations, while clinical scores are based on subjective evaluations, resulting in inconsistent feature semantics. Such heterogeneity may cause semantic misalignment and informational conflicts during feature fusion, making it difficult for the model to establish reliable cross-modal correspondences and ultimately weakening the effectiveness of multimodal integration.

**FIGURE 2:**
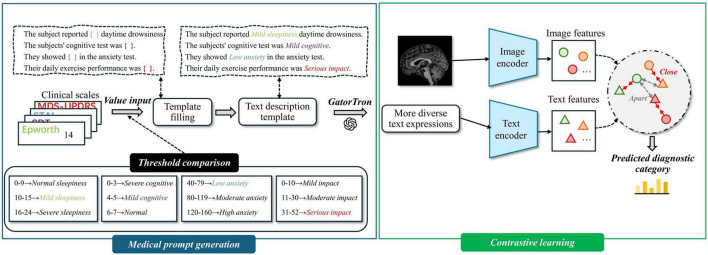
Overview of multimodal representation learning. Medical prompt generation takes clinical scales as input by converting structured data into textual prompts. It then integrates these prompts with magnetic resonance imaging (MRI) to learn more expressive multimodal representations.

To address these issues, we propose a multimodal representation learning (MRL) module, which incorporates contrastive learning to achieve cross-modal alignment. The MRL module consists of two components: medical prompt generation and contrastive learning. An overview of the MRL module is presented in Figure 2.

#### 3.3.1 Deriving medical prompts from clinical assessment scales

To enable semantic alignment between clinical rating scale data and MRI in a shared latent space and fully leverage their complementary modalities, we employ a large language model (LLM) to transform the original low-dimensional, discrete scale scores into high-dimensional, semantically enriched textual representations. This transformation mitigates the severe mismatch between clinical scores and MRI data in terms of feature dimensionality, informational content, and modality compatibility. Additionally, leveraging LLMs for sentence augmentation enhances the linguistic diversity of the textual inputs, thereby improving representational richness and information utilization.

We propose a framework for generating natural language descriptions from structured clinical rating scale data. The selected clinical assessments cover multiple symptom dimensions relevant to Parkinson’s disease, including motor performance, anxiety, cognition, and sleep disturbance—core aspects frequently used in clinical evaluations. The text generation process includes the following steps:

Step 1: Threshold Setting for Clinical Scales—Based on official reference manuals and expert recommendations (Goetz et al., 2008; Hazan et al., 2018; Knowles and Olatunji, 2020; Walker et al., 2020), we define category thresholds for continuous variables and map them to descriptive labels such as “Mild,” “Moderate,” “Significant,” and “Serious.”

Step 2: Variable Label Assignment—Each continuous score is categorized according to the predefined thresholds and assigned a corresponding text label that reflects symptom severity.

Step 3: Template Sentence Construction—For each clinical variable, a sentence template is designed [e.g., “The subject noted (ESS) drowsiness during the day.”], and placeholders are replaced with the assigned labels to generate interpretable and semantically clear descriptions.

Step 4: Textual Augmentation Using GatorTron (Yang et al., 2022)—Each template sentence is expanded into eight semantically consistent but lexically diverse variants using the GatorTron model.

Appendix A provides the pseudocode implementation of the aforementioned steps.

Template-based sentences only offer a single form of expressing clinical scale information, which may restrict the model’s ability to fully capture semantic meaning and limit its generalizability. Contrastive learning aims to maximize the similarity between positive sample pairs. If these pairs are limited to identical textual inputs, the model is constrained to a narrow semantic space.

To address this limitation, we leverage the GatorTron module to generate augmented text variants for each template sentence. Trained on large-scale clinical corpora, GatorTron is capable of producing semantically equivalent but lexically diverse clinical expressions. This diversity enhances the model’s ability to capture semantic similarity among conceptually related samples, thereby improving its capability to distinguish and generalize across similar instances. In addition, we manually reviewed a subset of representative augmented samples to ensure that the generated variants remained semantically consistent with the original clinical intent.

#### 3.3.2 Contrastive learning

**FIGURE 3:**
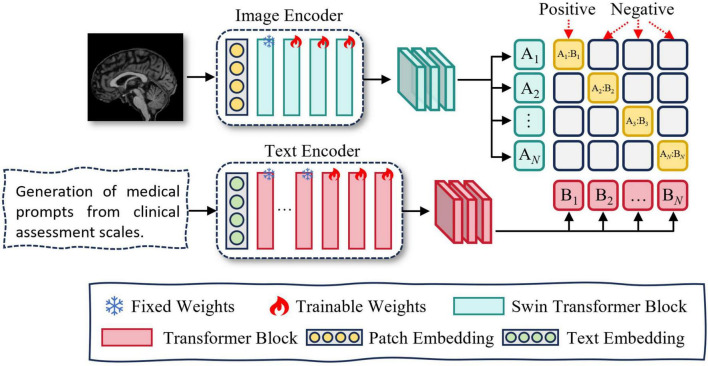
Overview of contrastive learning.

Directly fusing image and text modalities can lead to challenges such as heterogeneous feature distributions and semantic misalignment between modalities. To address these issues, we adopt contrastive learning to pretrain the text and image encoders. This approach encourages matched image–text pairs to be projected closer together, while pushing apart mismatched pairs in a shared embedding space. By learning modality-invariant representations, the encoders achieve cross-modal semantic alignment and better capture inter-modality correlations, thereby enhancing the effectiveness of multimodal fusion. An overview of the Contrastive learning module is presented in Figure 3. The specific training procedure is described as follows:

In this study, we utilize Swin UNETR as the image feature extractor. Swin UNETR is a Transformer-based encoder specifically designed for 3D medical imaging, consisting of a Patch Embedding layer and four hierarchical Swin Transformer Blocks. The Patch Embedding layer performs image partitioning and linear projection. The first Swin Transformer Block focuses on capturing low-level features such as edges and textures, which are generally task-invariant and thus do not require fine-tuning. The second to fourth blocks (stages 2–4) are responsible for modeling high-level semantic representations. Accordingly, we freeze both the Patch Embedding layer and the first Swin Transformer Block, while fine-tuning the remaining blocks (stages 2–4) to adapt to our downstream task.

We utilize PubMedBERT as the text feature extractor. PubMedBERT consists of a word embedding layer followed by 12 transformer blocks and is pretrained on a large corpus of biomedical literature from PubMed. The lower nine transformer layers are primarily responsible for capturing general linguistic representations, which are robust and domain-invariant, thus requiring no additional fine-tuning. In contrast, the upper three layers are more sensitive to task-specific contextual semantics. Accordingly, we freeze the first nine layers and fine-tune the top three layers to better adapt the model to our downstream task.

In this study, we pretrain the encoders via contrastive learning, with the objective of maximizing the similarity between positive pairs (i.e., matching text and image) while minimizing the similarity between negative pairs (i.e., mismatched text and image). Contrastive learning also serves as the foundation for achieving cross-modal feature alignment (Walker et al., 2020).

During mini-batch training, we define two types of contrastive loss terms: Text-to-Image Loss and Image-to-Text Loss. For the *b* sample in a mini-batch, the text input is denoted as *x*_*tb*_ and the image input as *x*_*ib*_.

The text input *x*_*tb*_ is processed by the text encoder *E*_*text*_ to extract the corresponding text feature vector as defined in Equation 1:


(1) $$f_{t\,b}=E_{t\,e\,x\,t}\,\left(x_{t\,b}\right)$$

Similarly, the image input *x*_*ib*_ is passed through the image encoder *E*_*img*_ to generate the corresponding image feature vector as defined in Equation 2:


(2) $$f_{i\,b}=E_{\mathrm{img}}\,\left(x_{i\,b}\right)$$

Let *f*_*tb*_ and *f*_*ib*_ denote the feature vectors of the text and image inputs, respectively. The temperature parameter τ controls the sharpness of the cosine similarity distribution in contrastive learning. A smaller τ emphasizes hard negatives but may cause training instability, while a larger τ improves stability at the potential cost of reduced feature discriminability. This study conducted ablation experiments on the τ values, with results demonstrating that τ = 0.1 achieves an optimal balance between training stability and model performance.

The text-to-image contrastive loss is defined as follows The text-to-image contrastive loss is defined as follows Equation 3:


(3) $${ℒ}_{t\to i}=\sum_{b=1}^{B}-\log\,\frac{\exp\,\left(\cos\,\left(f_{t\,b},f_{i\,b}\right)/\tau \right)}{\sum_{k=1}^{B}\exp\,\left(\cos\,\left(f_{t\,b},f_{i\,k}\right)/\tau \right)}$$

Similarly, the image-to-text contrastive loss is defined as follows Equation 4: for each image feature vector *f*_*ib*_ and its corresponding text feature vector *f*_*tb*_ as follows:


(4) $${ℒ}_{i\to t}=\sum_{b=1}^{B}-\log\,\frac{\exp\,\left(\cos\,\left(f_{\mathrm{ib}},f_{\mathrm{tb}}\right)/\tau \right)}{\sum_{k=1}^{B}\exp\,\left(\cos\,\left(f_{\mathrm{ib}},f_{\mathrm{tk}}\right)/\tau \right)}$$

The total contrastive loss is computed as follows Equation 5: the average of the two individual losses defined above:


(5) $$ℒ=\frac{1}{2}\,\left({ℒ}_{t\to i}+{ℒ}_{i\to t}\right)$$

By optimizing the above loss functions, the similarity between positive sample pairs is increased, encouraging them to be closer in the shared latent space. Conversely, the similarity between negative pairs is reduced, ensuring they remain well-separated. This process facilitates semantic alignment between the text and image feature representations.

### 3.4 Multi-modal interaction

In the task of Parkinson’s disease (PD) diagnosis, MRI images and medical prompt texts encode complementary dimensions of pathological information. MRI captures structural abnormalities in the brain (e.g., reduced signal intensity in the substantia nigra or, cortical atrophy), while medical prompts describe clinical symptoms (e.g., sleep disturbances, anxiety, cognitive decline). These two modalities exhibit inherent semantic correlations. To enhance their interaction, we introduce a cross-modal attention (CMA) module that reinforces semantic alignment between modalities. Specifically, symptom descriptions in the textual prompts guide the extraction of salient imaging features, helping to highlight PD-related brain regions. Simultaneously, the imaging data provide objective evidence to support clinical observations, thereby mitigating the subjectivity of rating scales. This bidirectional reinforcement improves both the accuracy and robustness of the diagnostic model: image features serve as objective references for textual descriptions, while text features provide semantic labels that facilitate clinical interpretation of neuroimaging changes. The detailed computation process is as follows:

Text-guided image enhancement is computed as follows Equation 6:


(6) $$I^{*}=\text{softmax}\,\left(\frac{Q_{1}\,K_{T}^{T}}{\sqrt{d}}\right)\,V_{T}$$

Here, the query, key, and value of image features are computed as follows Equation 7:


(7) $$Q_{I}=W_{Q}\,I,K_{I}=W_{K}\,I,V_{I}=W_{V}\,I$$

We use 8-head cross-modal attention and project both MRI and text features into a shared 512-dimensional space using linear layers. This ensures that the attention query, key, and value vectors are dimensionally aligned for stable interaction between modalities.

This bidirectional attention mechanism enables mutual enhancement: text semantics refine image representations (I*), while image-derived cues refine text embeddings (T*), forming a closed loop of cross-modal guidance. In this process, the semantic information provided by the textual features guides the image features to focus on brain regions associated with Parkinson’s disease, thereby improving the discriminative power of the image features.

Image-guided text enhancement is computed as follows Equation 8:


(8) $$T^{*}=\mathrm{softmax}\,\left(\frac{Q_{T}\,K_{I}^{T}}{\sqrt{d}}\right)\,V_{I}$$

Here, the query, key, and value of text features are computed as follows Equation 9:


(9) $$Q_{T}=W_{Q}\,T,K_{T}=W_{K}\,T,V_{T}=W_{V}\,T$$

In this process, the image features (K_I_,V_I_) serve as prior knowledge to constrain the text features (Q_T_), making them more consistent with the image modality, thereby reducing subjective errors in scoring.

Finally, the enhanced features I* and T* after fusion are obtained and used for the subsequent classification task to improve the diagnostic performance for Parkinson’s disease.

### 3.5 Classification

After the modality interaction step, the resulting MRI image features I* and medical prompt text features T* may still contain redundant or less informative components. Moreover, directly concatenating high-dimensional representations can lead to substantial computational overhead. To address this, we introduce an attention-based reduction module, which computes attention weights to adaptively select the most discriminative feature components, thereby enhancing generalization capability. We perform a self-attention operation on the fused features $\hat{F}$, as computed by the following Equations 10, 11:


(10) $$\alpha =\mathrm{softmax}\,\left(\frac{Q\,K^{T}}{\sqrt{d}}\right)$$

(11) $$\hat{F}=\sum_{i=1}^{m}\alpha_{i}\,V_{i}$$

QK^T^ ∈ ℝ^m ×m^ computes the pairwise similarity between the query and key vectors, reflecting the importance of each feature dimension. A scaling factor $\sqrt{d}$ is applied to prevent overly large values and ensure training stability. The attention weight matrix α ∈ ℝ^m ×m^, obtained via Softmax normalization, represents the relative importance of the features.

In the classification stage, we employ a two-layer fully connected network that takes the reduced-dimensional fused representation as input. The hidden layer uses ReLU activation to introduce nonlinearity and capture deeper semantic features, while the output layer applies the Softmax function to map feature scores into a categorical probability distribution, thereby estimating the likelihood that each sample belongs to a specific class based on multimodal inputs.

To further alleviate the effects of distributional discrepancies between different modalities in multimodal feature fusion, we introduce two complementary objective functions: a modality alignment loss (*L*_*alignment*_) to reduce inter-modal differences, and classification loss (*L*_*classification*_) to guide the final prediction task.

We use the cosine similarity loss function to define the modality alignment loss as follows Equation 12:


(12) $$L_{\mathrm{alignment}}=1-\frac{I^{*}\cdot T^{*}}{∥I^{*}∥\,∥T^{*}∥}$$

I*⋅ T* represents the dot product between the image feature vector I* and the text feature vector T*, which measures the directional similarity between the two.

∥ I* ∥∥ T* ∥ represents the product of the L2 norms (Euclidean norms) of the image feature vector I* and the text feature vector T*, which is used to normalize the result of the dot product.

We use the binary cross-entropy loss function as follows Equation 13:


(13) $$L_{\mathrm{classification}}=-\frac{1}{N}\,\sum_{i=1}^{N}\left[y_{i}\,\log\,\left(p_{i}\right)+\left(1-y_{i}\right)\,\log\,\left(1-p_{i}\right)\right]$$

where *N* denotes the total number of samples, *y*_*i*_ is the ground-truth label of the iii-th sample (1 for the positive class and, 0 for the negative class), and *p*_*i*_ is the predicted probability that the iii-th sample belongs to the positive class.

To balance the contribution of the alignment loss and the classification loss, we introduce a trade-off coefficient λ in the total loss function. The final loss is computed as follows Equation 14:


(14) $$ℒ=\lambda \cdot {ℒ}_{\mathrm{alignment}}+\left(1-\lambda \right)\cdot {ℒ}_{\mathrm{classification}}$$

We set λ = 0.54, which is empirically verified to achieve an optimal balance between cross-modal alignment and classification performance. The validation process is demonstrated in the ablation study section.

## 4 Experiments and results

### 4.1 Cross-validation

To ensure the robustness and reliability of the experimental results, and considering the size of the dataset, we employed five-fold cross-validation to evaluate the model’s performance.

The final average performance *M*_*k*_ is computed as follows Equation 15:


(15) $$M=\frac{1}{K}\,\sum_{k=1}^{K}M_{k}$$

To assess the stability of the results, we also computed the standard deviation of the performance across the five folds using the following equation as follows Equation 16:


(16) $$\sigma_{M}=\sqrt{\frac{1}{K}\,\sum_{k=1}^{K}{\left(M_{k}-M\right)}^{2}}$$

Furthermore, to avoid potential bias arising from imbalanced class distributions, we adopted stratified cross-validation, which ensures that the class proportions in each fold are consistent with those in the original dataset.

### 4.2 Experimental configuration

We employed the AdamW optimizer (Zhuang et al., 2022), setting the learning rate to 1 × 10^–5^, which is well-suited for training visual backbone models and Transformer-based architectures. The batch size was set to 128 to balance computational efficiency and training stability.

All experiments were conducted on a single Tesla V100 GPU, with mixed precision training enabled to optimize GPU memory usage and accelerate computation.

### 4.3 Evaluation metrics

To quantitatively evaluate the performance of PD diagnosis, three commonly used evaluation metrics were employed: accuracy (ACC), F1-score (F1), and the area under the receiver operating characteristic curve (AUC). The definitions of these metrics are as follows Equations 17, 18:


(17) $$\mathrm{Accuracy}=\frac{\mathrm{TP}+\mathrm{TN}}{\mathrm{TP}+\mathrm{TN}+\mathrm{FP}+\mathrm{FN}}$$

(18) $$\mathrm{Precision}=\frac{\mathrm{TP}}{\mathrm{TP}+\mathrm{FP}},\mathrm{Recall}=\frac{\mathrm{TP}}{\mathrm{TP}+\mathrm{FN}},F\,1=2\,\frac{\mathrm{Precision}\cdot \mathrm{Recall}}{\mathrm{Precision}+\mathrm{Recall}}$$

where the terms are defined as follows:

**TP**, true positive; **TN**, true negative; **FP**, false positive; **FN**, false negative.

### 4.4 Performance comparison

To justify the performance of our proposed ModFus-D for PD diagnosis using multi-modality data, in this study, we compared the performance of the proposed method with several competing approaches using the same dataset. The results are summarized in Table 2. A brief overview of these baseline methods is provided below.

**TABLE 2:** Performance comparison between MODFus-PD and comparative methods.

Models	ACC	AUC	F1
Concatenation	0.794 ± 0.31	0.869 ± 0.12	0.822 ± 0.07
DF	0.821 ± 0.21	0.837 ± 0.23	0.801 ± 0.11
LXMERT	0.864 ± 0.24	0.816 ± 0.15	0.776 ± 0.12
VilBERT	0.881 ± 0.08	0.925 ± 0.14	0.792 ± 0.31
ModFus-PD	0.903 ± 0.14	0.892 ± 0.12	0.840 ± 0.16

Feature Concatenation (Early Fusion): This is the most basic form of multimodal fusion, where MRI image features and medical prompt text features are directly concatenated at the feature extraction stage and then passed through a unified classifier for prediction.

Decision-Level Fusion (DF): Also known as late fusion, this approach first trains independent classifiers for each modality, and then fuses their outputs during the inference phase to make the final decision.

LXMERT: Originally developed for vision-and-language tasks, LXMERT employs a dual-stream Transformer architecture that encodes visual and textual inputs separately and achieves semantic alignment through cross-modal attention. In this study, LXMERT is adapted to capture semantic interactions between MRI scans and medical prompts, facilitating improved classification performance for PD diagnosis (Tan and Bansal, 2019).

ViLBERT: Based on BERT, ViLBERT adopts a dual-pathway Transformer design that supports the parallel encoding and cross-modal integration of heterogeneous modalities. While initially proposed for image-text matching, it is extended here to the medical domain to align features and jointly model MRI and medical prompt data, thereby enhancing diagnostic accuracy in Parkinson’s disease classification (Lu et al., 2019).

For fair comparison, LXMERT and ViLBERT were fine-tuned on the PPMI dataset using central axial MRI slices (224 × 224) and clinical prompts derived from score templates. Inputs were fed into the original model architectures. Training was conducted for 20 epochs using AdamW (1e-5, batch size 32) with binary cross-entropy loss and early stopping based on validation AUC.

ModFus-PD achieved superior performance compared to the baseline models in both classification accuracy (0.903) and F1-score (0.840), demonstrating the effectiveness of the proposed medical prompt generation and contrastive learning strategies. we calculated 95% confidence intervals for all models based on five-fold cross-validation. Results show that ModFus-PD outperforms all baselines with non-overlapping CIs in at least two of the three metrics (ACC, AUC, F1), supporting the statistical significance of its improvements.

For the AUC metric, ModFus-PD slightly underperformed VilBERT (0.892 vs. 0.925), which may be attributed to VilBERT’s parallel encoder architecture and cross-attention mechanism, well-suited for modeling global cross-modal dependencies. This advantage is particularly evident in scenarios where modality distributions are relatively balanced, enabling more comprehensive integration and thus improved AUC performance.

The ACC of the decision-level fusion method (DF) reached 0.821, which is lower than that of feature concatenation (0.794), suggesting that fusing independent model predictions at the decision stage may be insufficient to capture cross-modal complementarities, leading to suboptimal performance in PD diagnosis.

### 4.5 Ablation experiment

To better understand the contributions of different components, we conducted the following three ablation studies: (1) We compared the performance of models using a direct fusion of numerical scale values and imaging features with those using text-converted scales, to assess the necessity of transforming structured numerical data into medical text prompts. (2) We examined the effect of using different individual scales and scale combinations to determine which configuration most effectively enhances model performance. (3) We evaluated how the number of augmented sentences generated during the text enhancement process influences the final classification performance.

#### 4.5.1 Evaluating the effectiveness of the clinical scale-to-text conversion module

In this experiment, we directly used the original clinical scale data in structured tabular format along with 3D MRI data to train the model, by passing the intermediate step of converting numerical values into textual descriptions. The goal was to examine whether directly leveraging numerical clinical scores alongside imaging features can yield competitive performance in distinguishing Parkinson’s disease patients from healthy controls, and to assess the impact of clinical score-to-text conversion on classification accuracy. The results are summarized in Table 3.

**TABLE 3:** Comparison of performance between tabular data + MRI and text data + MRI.

Mode	ACC	AUC	F1
Tabular data + MRI	0.874 ± 0.17	0.867 ± 0.35	0.793 ± 0.14
Text data + MRI	0.903 ± 0.14	0.892 ± 0.12	0.840 ± 0.16

In terms of classification accuracy, the combination of generated clinical Tabular data + MRI and MRI features (Text + MRI) achieved an accuracy of 0.903, representing a modest improvement over the Tabular data + MRI setting (0.874). This suggests that enhancing the semantic richness of scale data through text generation can improve the model’s ability to integrate and classify multimodal inputs. Interestingly, for the AUC metric, Tabular data + MRI + MRI yielded a slightly higher score (0.892 vs. 0.860), indicating that the model using raw tabular inputs may exhibit stronger robustness across varying classification thresholds. However, in terms of F1-score, the Text + MRI setting significantly outperformed Tabular data + MRI + MRI (0.840 vs. 0.793), demonstrating ModFus-PD’s superior balance between precision and recall, particularly in capturing features of the positive (patient) class.

Clinical scale data are typically represented in discrete numerical format, while MRI scans encode high-dimensional continuous spatial information. The direct concatenation of such heterogeneous modalities can lead to poor alignment and limited cross-modal interaction. By transforming structured numerical inputs into natural language descriptions, the textual representations become semantically aligned with high-level visual features extracted from MRI, thereby facilitating cross-modal alignment.

We further applied contrastive learning to pre-train the text and image encoders, encouraging both modalities to align in a shared embedding space and enhancing the complementarity between them. Additionally, we implemented a text augmentation strategy by converting each clinical scale record into a template sentence, and then generating multiple semantically equivalent descriptions. This not only enriches the model’s understanding of clinical symptoms but also effectively augments the training set, mitigating the challenge of data scarcity—a common issue in medical AI tasks.

#### 4.5.2 Analysis of the impact of different clinical scale combinations on model performance

In this experiment, we investigated how various combinations of four clinical rating scales affect classification performance within the text–MRI multimodal framework for Parkinson’s disease diagnosis. The selected scales encompass four key dimensions: motor function (MDS-UPDRS), anxiety levels (STAI), sleep quality (ESS), and cognitive function (CDT). From a clinical perspective, in this study, we aimed to assess the relative diagnostic contribution of each dimension and determine which combinations most effectively support accurate PD classification. We implemented two primary experimental configurations:

(1) Single-modality baseline

**FIGURE 4:**
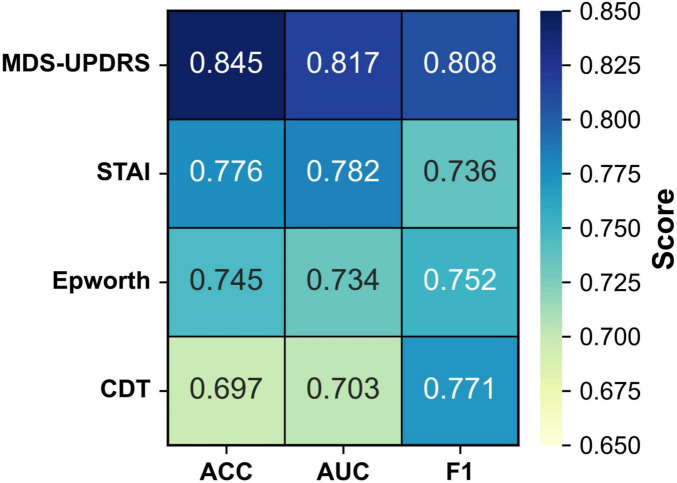
Heatmap of model performance using different single scales.

To evaluate the individual diagnostic value of each clinical scale, we conducted four single-scale experiments. In each experiment, the model was trained and tested using only the corresponding clinical scale in combination with MRI data. This setting enables a direct comparison of the classification performance contributed by each scale, thereby quantifying its standalone diagnostic utility and providing a reference for subsequent multi-scale fusion strategies. The results are summarized in Figure 4 and Table 4.

**TABLE 4:** Comparison of model performance using different single scales.

	ACC	AUC	F1
MDS-UPDRS	0.845 ± 0.07	0.817 ± 0.12	0.808 ± 0.13
STAI	0.776 ± 0.14	0.782 ± 0.23	0.736 ± 0.11
ESS	0.745 ± 0.28	0.734 ± 0.18	0.752 ± 0.12
CDT	0.697 ± 0.17	0.703 ± 0.17	0.771 ± 0.15

As illustrated in Table 3, when each clinical scale was evaluated individually, the motor function scale (MDS-UPDRS) demonstrated the highest diagnostic performance, with an accuracy of 0.845, an AUC of 0.817, and an F1 score of 0.808. These results suggest that motor-related symptoms offer the most discriminative information for identifying Parkinson’s disease.

In contrast, when using the CDT scale—which assesses cognitive function—alone, the performance dropped to 0.697 (ACC), 0.703 (AUC), and 0.771 (F1). This may be attributed to the fact that cognitive impairments typically manifest in the middle-to-late stages of PD, and thus may not be apparent in early-stage patients, limiting the effectiveness of CDT as a standalone diagnostic indicator.

(2) Multi-scale combination performance

**FIGURE 5:**
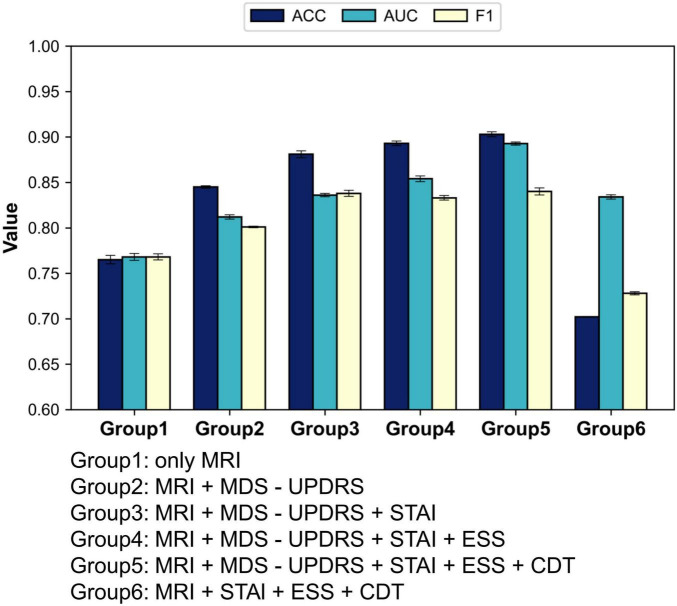
Performance changes with the sequential addition of individual scale models.

We first adopted the image-only model as a baseline and then sequentially incorporated the four clinical scales—MDS-UPDRS, STAI, ESS, and CDT—to evaluate the incremental benefit of each additional modality. With this experiment, we aimed to assess how different scale combinations contribute to classification accuracy and to identify the most effective fusion strategy for PD diagnosis. The results are summarized in Figure 5 and Table 5.

**TABLE 5:** Performance changes with the sequential addition of individual scale models.

MRI	MDS-UPDRS	STAI	ESS	CDT	ACC	AUC	F1
√					0.765 ± 0.07	0.768 ± 0.18	0.768 ± 0.08
√	√				0.845 ± 0.14	0.812 ± 0.37	0.801 ± 0.06
√	√	√			0.881 ± 0.21	0.836 ± 0.08	0.838 ± 0.08
√	√	√	√		0.893 ± 0.18	0.854 ± 0.11	0.833 ± 0.15
√	√	√	√	√	0.903 ± 0.08	0.8924 ± 0.17	0.840 ± 0.09
√		√	√	√	0.702 ± 0.04	0.834 ± 0.23	0.728 ± 0.17

As illustrated in Table 4, the model’s classification performance improved progressively with the inclusion of additional clinical scales. The most substantial gain was observed with the introduction of MDS-UPDRS, while CDT contributed a modest yet noticeable improvement.

These findings consistently highlight the pivotal role of MDS-UPDRS in the diagnostic task. To further assess its impact, we conducted an experiment excluding MDS-UPDRS and retaining only STAI, ESS, and CDT. The model achieved an accuracy of merely 70.2%, which was even lower than that obtained using any single scale alone.

One possible explanation is that the non-motor scales may contain conflicting or redundant information. Although STAI, ESS, and CDT each capture different aspects of a patient’s mental and physiological state, they may lack strong complementarity. Instead, overlapping content, measurement noise, or semantic inconsistencies could arise.

For instance, one patient may present with high anxiety (high STAI score) but no signs of sleepiness (normal ESS score), while another may show the opposite pattern. In the absence of motor symptom information, such variation may prevent the model from learning stable and discriminative decision boundaries.

These inconsistencies across non-motor scales may ultimately hinder feature coherence, resulting in diminished classification performance when these scales are fused without MDS-UPDRS.

#### 4.5.3 Analysis of the impact of different clinical scale combinations on model performance

With this experiment, we investigated the effect of the number of augmented sentences (i.e., the number of sentences expanded from each template sentence) on model performance, in order to determine the optimal amount of textual augmentation. The model’s performance under different numbers of augmented sentences N was compared, where *N* = 1 indicates using only the template sentence, and *N* = 4, *N* = 8, *N* = 16, and *N* = 32 indicate increasing levels of augmentation.

**FIGURE 6:**
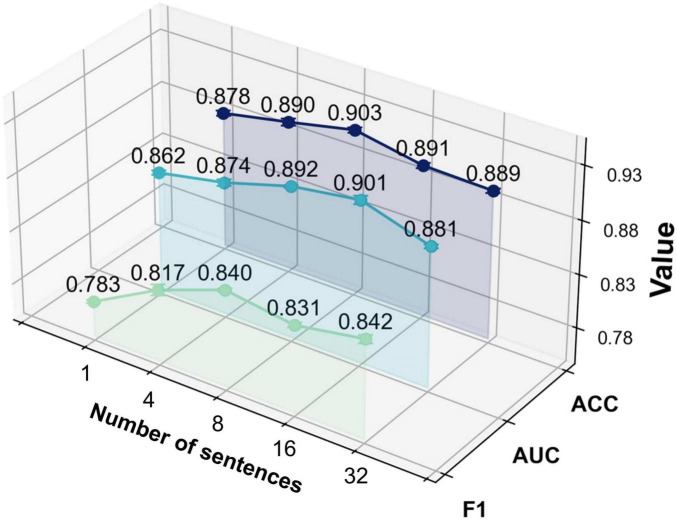
The impact of text enhanced sentence expansion on model performance.

As shown in Figure 6 and Table 6, the model yields the lowest performance when *N* = 1 (i.e., using only the template sentence), significantly underperforming compared to configurations with augmented text. When *N* = 4, classification performance improved, suggesting that a moderate degree of text augmentation increases the semantic diversity of positive samples and enhances the model’s ability to distinguish between classes. At *N* = 8, the model achieved peak accuracy. However, further increasing the number of augmented sentences (*N* = 16; *N* = 32) led to a slight improvement in AUC and F1-score, while accuracy began to decline.

**TABLE 6:** The impact of text enhanced sentence expansion on model performance.

N	ACC	AUC	F1
1	0.878 ± 0.07	0.862 ± 0.21	0.783 ± 0.15
4	0.890 ± 0.11	0.874 ± 0.31	0.817 ± 0.11
8	0.903 ± 0.14	0.892 ± 0.12	0.840 ± 0.16
16	0.891 ± 0.09	0.901 ± 0.09	0.831 ± 0.22
32	0.889 ± 0.19	0.881 ± 0.08	0.842 ± 0.18

These results suggest that the ModFus-PD framework benefits from limited text augmentation in enhancing discriminative capability. Beyond a certain point, however, excessive augmentation may introduce noisy or semantically inconsistent samples, blurring the alignment between textual prompts and corresponding MRI features, thereby impairing the formation of clear positive and negative pairs during contrastive learning.

Nevertheless, the slight continued gains in AUC and F1 -score with larger N indicate that, in some scenarios, additional augmented data may still contribute to improved class separability. Therefore, when computational resources are constrained, it is essential to balance performance gains with the associated computational cost to identify an optimal augmentation strategy.

#### 4.5.4 Parameter ablation

(1) Effect of loss weight λ on cross-modal fusion

To explore the influence of balancing alignment and classification objectives in our multimodal diagnostic framework, we conducted experiments by varying the loss weight coefficient λ in the total loss formulation:

$$ℒ=\lambda \cdot {ℒ}_{\mathrm{alignment}}+\left(1-\lambda \right)\cdot {ℒ}_{\mathrm{classification}}$$

**FIGURE 7:**
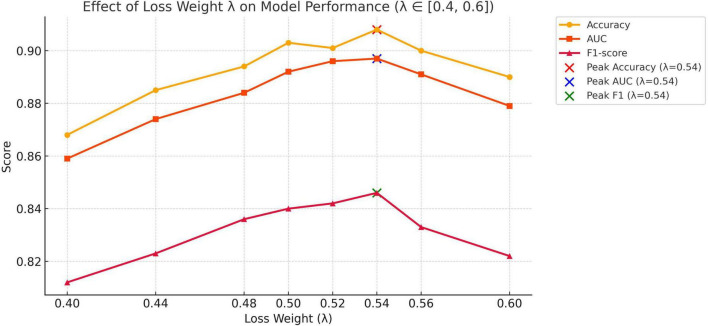
Effect of loss weight λ on classification performance.

The results, summarized in Table 7 and illustrated in Figure 7, show that model performance improves as λ increases from 0.40 and peaks at λ = 0.54, with noticeable gains in accuracy, AUC, and F1-score. These findings confirm that moderately increasing the influence of the alignment objective leads to more effective fusion of image and text modalities. The choice of λ = 0.54 in our final model is thus empirically justified and reflects a well-calibrated balance between representation alignment and task-specific supervision.

**TABLE 7:** Effect of loss weight λ on classification performance.

λ	Accuracy	AUC	F1-score
0.40	0.868	0.859	0.812
0.44	0.885	0.874	0.823
0.48	0.894	0.884	0.836
0.50	0.901	0.882	0.837
0.52	0.900	0.890	0.839
**0.54**	**0.903**	**0.892**	**0.840**
0.56	0.900	0.891	0.833
0.60	0.890	0.879	0.822

The bold values in the table indicate the optimal performance metrics.

(2) Effect of learning rate and weight decay on optimization performance

To determine appropriate values for the learning rate and weight decay coefficient, we conducted grid search experiments within the following ranges, which were selected based on commonly used settings for fine-tuning Transformer-based architectures reported in prior literature:

Learning Rate: {1 × 10^–5^, 5 × 10^–5^, 1 × 10^–4^, 5 × 10^–4^}

Weight Decay: {1 × 10^–6^, 1 × 10^–5^, 1 × 10^–4^}

**FIGURE 8:**
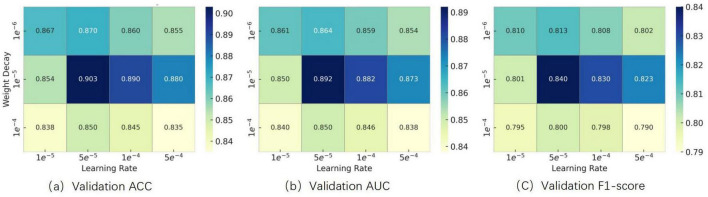
Effect of learning rate and weight decay on optimization performance.

We systematically evaluated combinations of these hyperparameters using a grid search strategy. Experimental results showed that setting both values to 1 × 10^–5^ yielded the best validation performance in terms of accuracy, AUC, and F1-score. The results are illustrated in Figure 8. Therefore, this configuration was adopted in all subsequent experiments.

(3) Effect of CMA layer depth on modality interaction

To evaluate the impact of cross-modal attention depth, we conducted ablation experiments with 1, 2, and 3 stacked Cross-Modal Attention (CMA) layers. As summarized in Table 8, increasing the depth to 2 layers slightly improved the accuracy (from 0.903 to 0.905), but resulted in noticeable declines in AUC (from 0.892 to 0.878) and F1-score (from 0.840 to 0.831). Further increasing the depth to 3 layers slightly recovered F1-score (0.832) but still underperformed the single-layer model overall. These results suggest that deeper attention layers may introduce redundancy and degrade the model’s ability to maintain class-wise balance. To ensure robustness and efficiency, we adopt a single CMA layer in the final model configuration.

**TABLE 8:** Effect of cross-modal attention (CMA) depth on model performance.

CMA depth (layers)	ACC	AUC	F1-score
**1**	**0.903**	**0.892**	**0.840**
2	0.905	0.878	0.831
3	0.902	0.882	0.832

The bold values in the table indicate the optimal performance metrics.

## 5 Discussion

**FIGURE 9:**
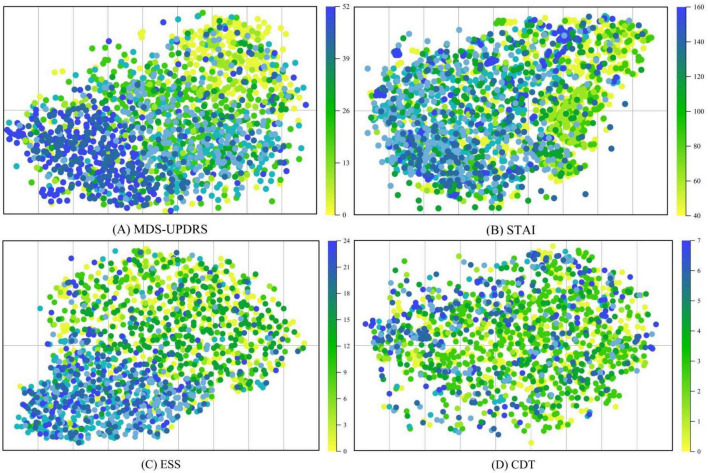
t-SNE was used to visualize MRI representations generated by the trained image encoder. The visualization includes four clinical assessment scales used during model training: **(A)** The movement disorder society-unified Parkinson’s disease rating Scale (MDS-UPDRS). **(B)** The Epworth sleepiness scale (ESS). **(C)** The state-Trait Anxiety Inventory (STAI). **(D)** The clock drawing test (CDT).

In this section, we visualize the MRI embeddings extracted by the trained image encoder using t-SNE decomposition. As shown in Figure 9 the resulting low-dimensional feature spaces are color-coded according to individual clinical rating scales, namely, MDS-UPDRS, ESS, STAI, and CDT, allowing us to assess the degree to which the image encoder captures scale-specific distinctions. Despite converting continuous clinical variables into categorized textual prompts, the ModFus-PD image encoder was still able to learn semantically

meaningful feature representations, particularly in association with three scales: (1) MDS-UPDRS, (2) STAI, and (3) ESS.

The experimental results on the PPMI dataset demonstrate that ModFus-PD outperforms several competitive methods. These results validate the critical importance of leveraging cross-modal complementarity in complex medical tasks—especially for diseases like Parkinson’s, where clinical symptoms and MRI features are highly interdependent.

The t-SNE visualization results in Figure 9 demonstrate that MRI-derived features exhibit a well-structured distribution in the reduced-dimensional space. Notably, features associated with MDS-UPDRS scores form distinct clusters, indicating that the MRI representations effectively capture motor symptom patterns and are well aligned with MDS-UPDRS annotations in the shared multimodal space.

**FIGURE 10:**
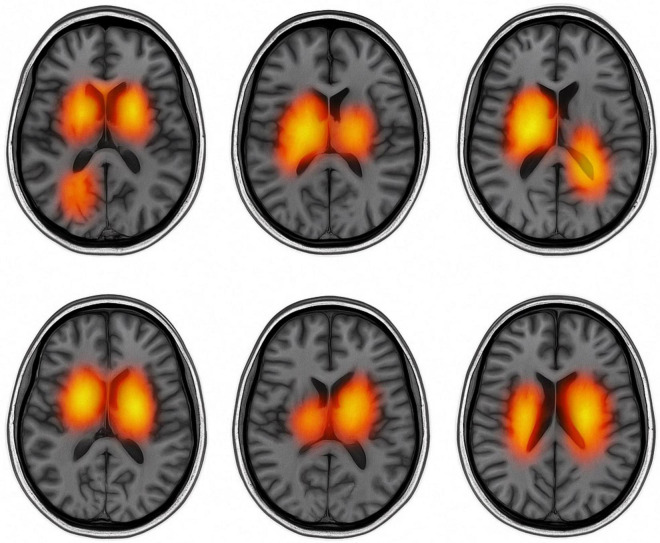
Text-guided attention heatmaps on axial MRI slices from six subjects.

Figure 10 presents text-guided attention heatmaps on axial MRI slices from six representative subjects, illustrating the spatial focus of the proposed cross-modal diagnostic model. The highlighted regions reflect areas where the model attends most when guided by clinical symptom prompts. Notably, the attention consistently concentrates on Parkinson’s disease-relevant regions such as the basal ganglia, substantia nigra, and motor cortex, supporting the model’s ability to align semantic information with neuroanatomical pathology.

These findings further validate the effectiveness of multimodal representation learning. The contrastive pretraining strategy enables the encoder to establish a unified cross-modal embedding space, where MRI and clinical scale data are meaningfully aligned, thereby enhancing the robustness of PD diagnosis. It is important to note that the effectiveness of modality alignment is largely influenced by the degree of association between clinical scale information and the pathological features captured by MRI. Among the evaluated scales, MDS-UPDRS achieves the most robust alignment, likely due to its strong correlation with motor-related structural abnormalities observable in MRI scans.

In contrast, CDT exhibits weaker alignment performance, as cognitive impairments assessed by this scale are more challenging to represent accurately through structural imaging alone.

Despite promising results, this study has several limitations. The model is trained and evaluated solely on the PPMI dataset, which may limit generalizability to broader clinical populations. Future work will focus on validating the model in multicenter settings and enhancing its robustness across diverse clinical scenarios.

From a computational perspective, ModFus-PD introduces a certain degree of overhead, particularly in the contrastive learning pretraining and cross-modal attention stages. Processing a single 3D MRI input involves approximately 120 GFLOPs, and the average training time is approximately 9 min per epoch (batch size = 4) on a Tesla V100 GPU, totaling around 8 h for 50 epochs. The contrastive learning stage incurs additional cost due to dual-stream encoding and similarity computation, while the cross-modal attention module increases complexity through feature alignment between modalities. Despite these demands, inference per subject remains efficient at approximately 0.4 s, supporting clinical usability. Overall, although the computational cost is not minimal, it is justified by the substantial performance improvements in accuracy, AUC, and F1-score. Future work may explore lightweight designs to further improve deployment feasibility in real-world healthcare settings.

## 6 Conclusion

In this study, we proposed ModFus-PD, a multimodal diagnostic framework for early-stage Parkinson’s disease (PD), which integrates MRI data and clinical rating scales. Given the inherent distributional differences between MRI and structured clinical data, our multimodal representation learning module is designed to align features from both modalities within a shared embedding space. Both the image and text encoders are pretrained using contrastive learning to ensure high-quality feature extraction for downstream tasks such as cross-modal interaction and classification. To effectively leverage the complementary nature of imaging and clinical data, we introduced a cross-attention mechanism that enables the model to dynamically exchange information between the MRI and textual modalities. This facilitates richer cross-modal interactions and allows the final classifier to make more informed predictions by combining both structural and clinical cues. Experimental results on the PPMI dataset show that ModFus-PD consistently outperforms several competitive baselines. These findings underscore the importance of modeling cross-modal complementarity in complex medical tasks, particularly for conditions like PD where clinical manifestations are closely tied to neuroanatomical changes observable in MRI.

## Data Availability

The raw data supporting the conclusions of this article will be made available by the authors, without undue reservation.

## Appendix

**Appendix A:** Algorithm: Deriving medical prompts from clinical scales

**Input:** Clinical assessment scale scores, template sentences, and corresponding thresholds. **Output:** Expanded sentence variations **1 For each** clinical assessment scale: **2** Define threshold mappings: **3** THRESHOLDS[scale] ← [(lower1, upper1, label1), (lower2, upper2, label2),…] **4 End For** **5 If** scale_name is not in THRESHOLDS: **6** Return “Unknown Scale” **7 For each** (lower, upper, label) in THRESHOLDS[scale_name]: **8 If** lower ≤ score ≤ upper: **9 Return** label **10 End For** **11 Return** “Unknown Score” **12** Define template sentences: **13** TEMPLATES ←{ **14 “**MDS-UPDRS”: “The subject’s MDS-UPDRS assessment indicates {label}.” **15 “**STAI”: “The subject reports {label} anxiety based on the STAI scale.” **16** “ESS”: “The subject noted {label} drowsiness during the day.” **17 “**CDT”: “|The cognitive assessment places the subject in the {label} category.” **18** } **19 If** scale_name is in TEMPLATES: **20** Replace {label} in TEMPLATES[scale_name] with assigned label **21 Return** the generated sentence **22 Else**: **23 Return** “No template available for this scale.” **24** Create an empty list for sentence variations **25 For** i from 1 to 8: **26** Append “(Variation i)” to the sentence **27 End For** **28 Return** the list of variations **29** For each (scale, score) in [(“MDS-UPDRS,” 15), (“STAI,” 100), (“ESS,” 12), (“CDT,” 5)]: **30** Generate base sentence using the assigned label **31** Expand the base sentence into multiple variations **32** Output base sentence and expanded variations **33 End For**
